# Supplementary material for: Epigenetic Landscape Is Largely Shaped by Diversiform Transposons in Aegilops tauschii
Source: Int J Mol Sci. 2023 May 27;24(11):9349. doi: 10.3390/ijms24119349 (PMC10253722; doi:10.3390/ijms24119349)
Supplement: Supplementary file 1 [file ijms-24-09349-s001.zip › ijms-2378947-supplementary.pdf]

Table S1. List and catalog of histone antibodies used in this study.

No.	Antibody	Source	Catalog N°	Host/Isotype	Clonality	Format
1	CENH3	CWBio	Zhang lab	Rabbit IgG	Polyclonal	Serum
2	H2Bub	Médimabs	MM-0029	Mouse IgG2a	Monoclonal	Purified
3	H3	Abcam	ab1791	Rabbit IgG	Polyclonal	Purified
4	H3K18ac	Abcam	ab1191	Rabbit IgG	Polyclonal	Purified
5	H3K27ac	Millipore	07-360	Rabbit IgG	Polyclonal	Serum
6	H3K27me1	Millipore	07-448	Rabbit IgG	Polyclonal	Purified
7	H3K27me2	Abcam	ab24684	Rabbit IgG	Polyclonal	Purified
8	H3K27me3	Millipore	07-449	Rabbit IgG	Polyclonal	Purified
9	H3K36me2	Abcam	ab9049	Rabbit IgG	Polyclonal	Purified
10	H3K36me3	Abcam	ab9050	Rabbit IgG	Polyclonal	Purified
11	H3K4me1	Millipore	07-436	Rabbit IgG	Polyclonal	Purified
12	H3K4me2	Millipore	07-030	Rabbit IgG	Polyclonal	Serum
13	H3K4me3	Millipore	07-473	Rabbit IgG	Polyclonal	Purified
14	H3K56ac	Abcam	ab71956	Rabbit IgG	Polyclonal	Purified
15	H3K9ac	Millipore	07-352	Rabbit IgG	Polyclonal	Serum
16	H3K9me2	Abcam	ab1220	Mouse IgG2a	Monoclonal	Purified
17	H3K9me3	Abcam	ab8898	Rabbit IgG	Polyclonal	Purified
18	H4K20me1	Millipore	07-748	Rabbit IgG	Polyclonal	Purified
19	H4K5ac	Millipore	07-327	Rabbit IgG	Polyclonal	Serum

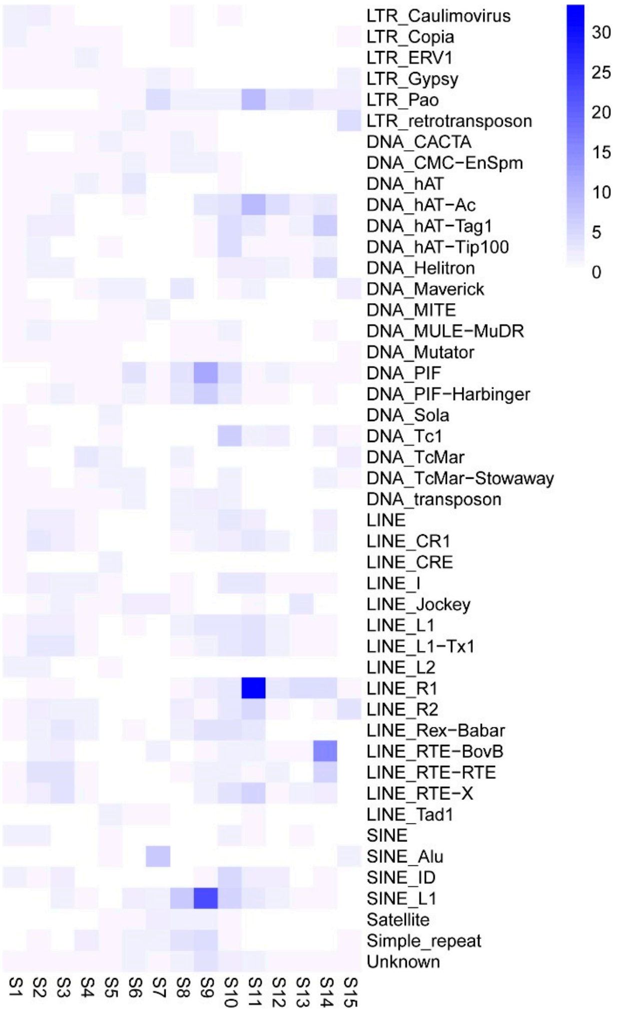

Figure S1. The heatmap represents the fold enrichments of TEs of different superfamilies in chromatin states.

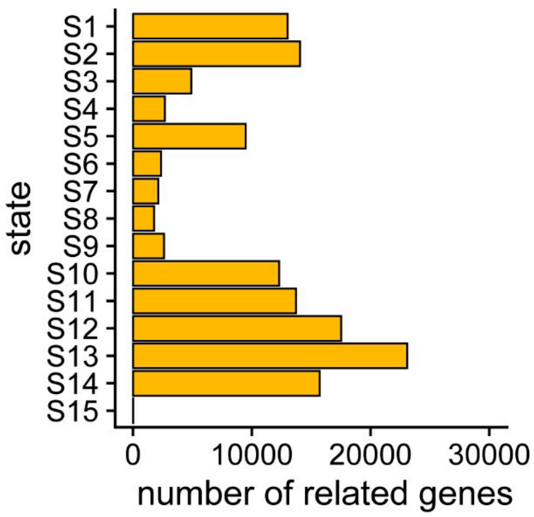

Figure S2. The bar chart represents the number of related genes in the chromatin states.

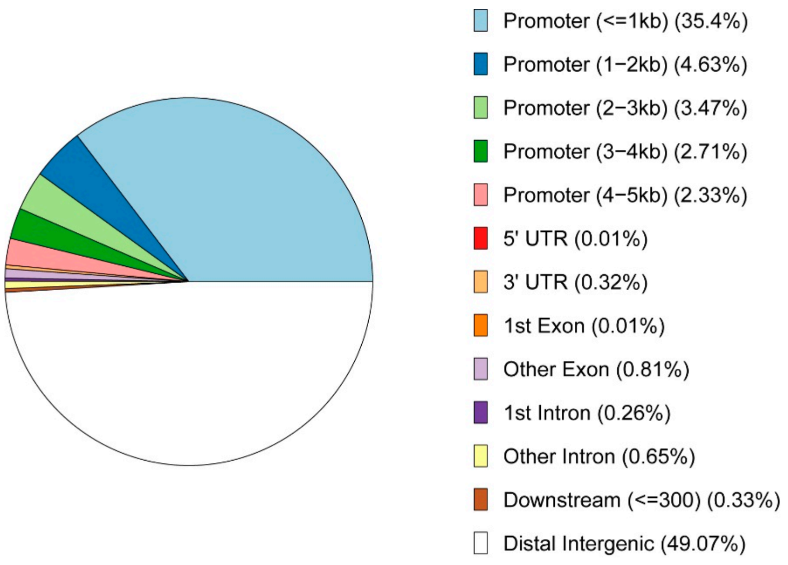

Figure S3. The pie chart shows the distribution of MNase HSs in the AL8/78 genome.

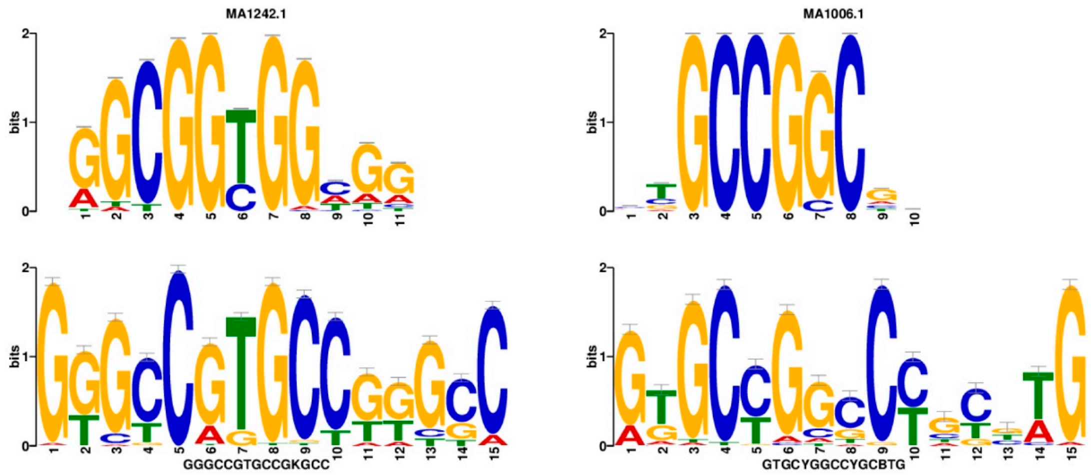

Figure S4. The diagrams show similarities between the known transcription factor-binding motifs of the ERF/DREB family in *Arabidopsis* (upper diagram) and the motifs found in hAT-Ac TE-HSs in *Ae. tauschii* (lower diagram). The motif similarities were searched and quantified by Tomtom software from JASPAR (NON-REDUNDANT) CORE plants database (Wasserman and Sandelin, 2004). The motifs in *Arabidopsis* were previously reported by O'Malley et al., 2016 and Weirauch et al., 2014.

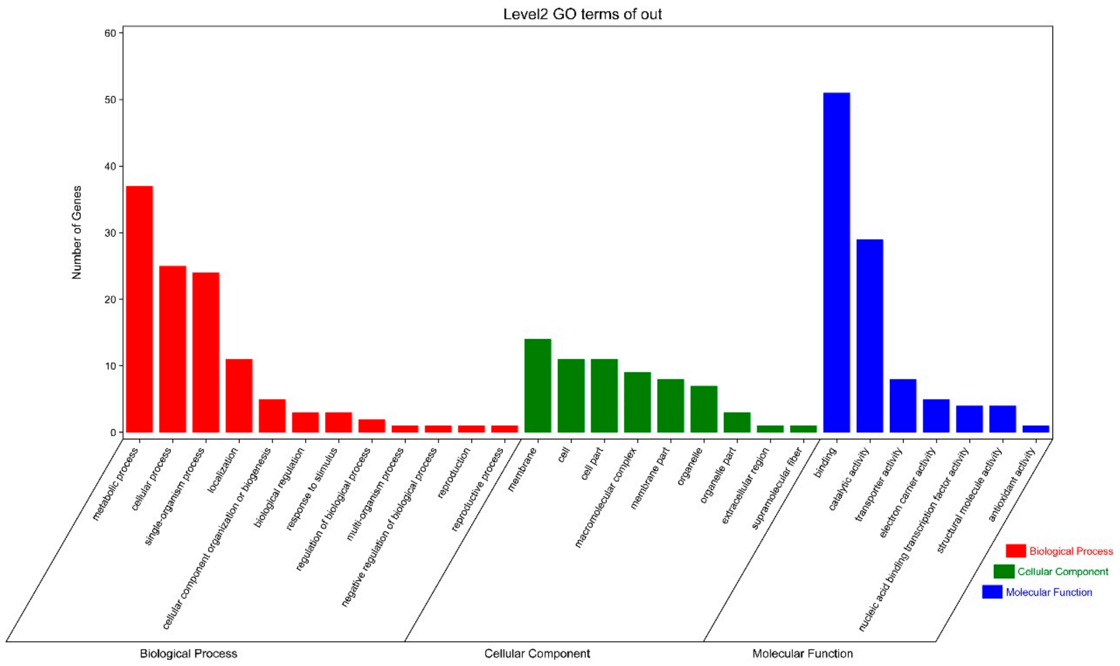

Figure S5. The top enriched GO terms of proximal TE-HS related genes.

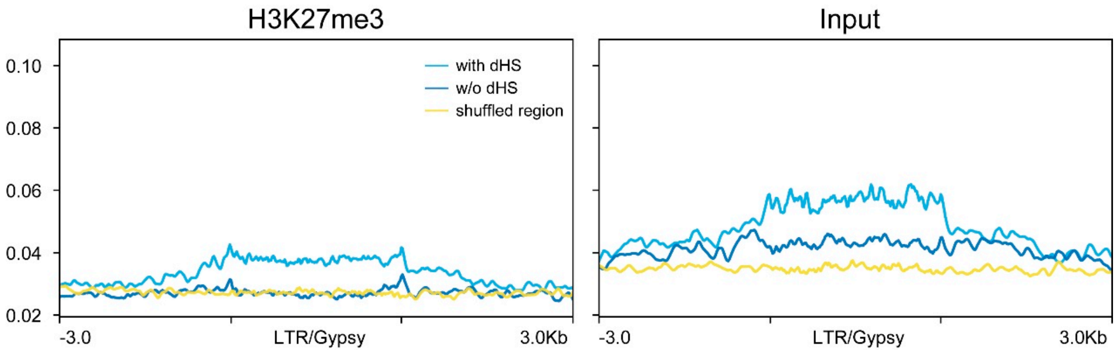

Figure S6. The profiles show H3K27me3 and Input distributions around LTR/Gypsy with or without dHS. Shuffled genomic regions are used as control.

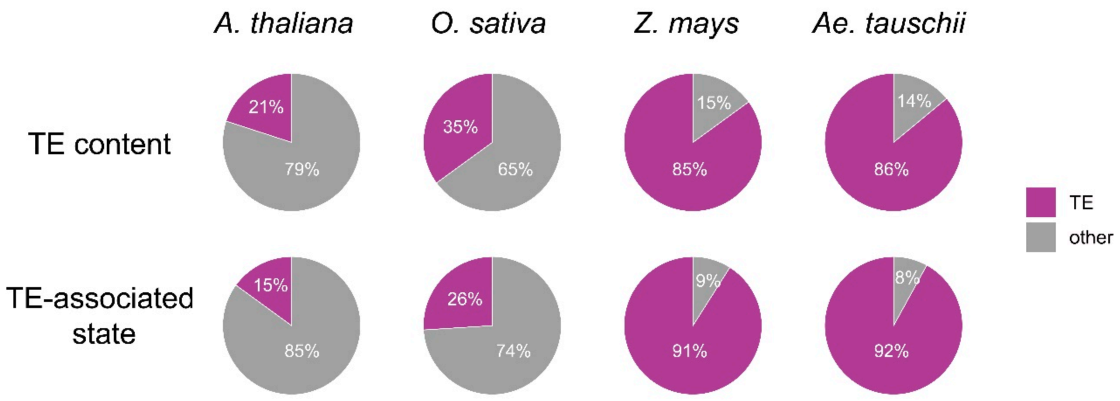

Figure S7. The pie charts show the percentage of TEs in the genome (top) and the percentage of TE-associated chromatin states in all chromatin states (bottom) in *Arabidopsis thaliana*, *Oryza sativa*, *Zea mays*, and *Aegilops tauschii*, respectively. The chromatin state data used in this study for *A. thaliana*, *O. sativa*, and *Z. mays* was obtained from the Plant Chromatin State Database (PCSD) constructed by Liu et al., 2018, a study collected public and in-house plant epigenomic data sets and applied a Hidden Markov Model to define chromatin states.
